# Pleural Rosai-Dorfman disease complicated with renal clear cell carcinoma: a case report and literature review

**DOI:** 10.3389/fonc.2024.1476243

**Published:** 2025-01-07

**Authors:** Sudan Tang, Xinju Yang, Shunan Wang, Qin Xiao

**Affiliations:** ^1^ Department of Radiology, Daping Hospital, Army Medical University, Chongqing, China; ^2^ Chongqing Clinical Research Centre of Imaging and Nuclear Medicine, Chongqing, China; ^3^ Department of Neurology, The Second People's Hospital of Banan District, Chongqing, China

**Keywords:** rosai-dorfman disease, pleura, computed X-ray tomography, renal clear cell carcinoma, pathology

## Abstract

**Background:**

Rosai-Dorfman disease (RDD), also known as sinus histiocytosis with massive lymphadenopathy, is a rare non-malignant disorder characterized by excessive proliferation of histiocytes, the cause of which remains unknown. Although the lymph nodes are the most commonly affected site, some patients may present with extranodal involvement, particularly in the skin, nasal cavity, eyes, and bones. In this report, we aim to present a unique case of RDD with pleural involvement in a 61-year-old patient. To our knowledge, only a few cases of RDD primarily manifesting as pleural disease have been reported thus far.

**Case summary:**

A 61-year-old male with left brachial plexus neuropathy was found to have a space-occupying lesion in the left posterior pleura. Initial chest CT showed a 5.7cm × 1.3cm soft tissue density shadow, which grew to 6.8cm × 1.7cm two years later. Given a history of clear cell renal cell carcinoma, pleural metastasis was suspected. The patient declined a needle biopsy but underwent surgical excision. Postoperative pathology and immunohistochemistry confirmed Rosai-Dorfman disease (RDD). After surgery, the patient received anti-infection, phlegm reduction, rehydration, and analgesia treatments and recovered well.

**Conclusion:**

RDD originating in pleura is rare, its clinical and imaging manifestations lack specificity, and it is easy to be misdiagnosed. It is crucial to consider the possibility of RDD when encountering nodular pleural masses or lamellar soft tissue lesions. Differential diagnosis should include pleural mesothelioma, pleural metastases, solitary fibrous neoplasms, and schwannomas of the pleura. Ultimately, a definitive diagnosis can be achieved through pathology and immunohistochemistry analysis.

## Introduction

Rosai-Dorfman disease (RDD) is a rare histiocytic disease that was initially described by Paul Destombes in 1965 and later identified by Rosai and Dorfman in 1969 as “sinus histiocytosis with massive lymphadenopathy” (1–3). The exact cause of RDD is still unknown, although some studies suggest a potential association with viral and bacterial infections, as well as immune system disorders (4). The disease primarily affects children and young adults, with a higher prevalence in males. Rosai-Dorfman disease can be classified into three types based on the extent of lesion involvement: lymph node, extranodal, and mixed; with lymph node involvement being the most common. Among the extranodal presentations, skin involvement is the most frequently observed. While Rosai-Dorfman disease involving the chest is rare, pulmonary manifestations, such as bronchial lumen polyposis causing airway obstruction, as well as mediastinal and hilar lymph node lesions resembling sarcoidosis, are the most commonly reported (5, 6). Here, we present a rare case of RDD exclusively involving the pleura, representing an unusual extranodal manifestation.

## Case presentation

A 61-year-old male patient presented at the Army Specialty Medical Center with a left posterior pleural space-occupying lesion that had been present for over two years. The patient had not received any treatment during this time. When the patient was admitted to the Army Specialty Medical Center over 2 years ago with a diagnosis of “left brachial plexus neuropathy”, a chest CT scan revealed a mass in the left pleural cavity. However, no further investigation was conducted at that time. Currently, the patient is experiencing mild chest tightness after physical activity without any clear trigger, along with fatigue. These symptoms have persisted for over a month. As a result, the patient sought medical attention again at the Army Special Medical Center. The patient underwent a resection for left renal clear cell carcinoma one year ago. Additionally, the patient underwent internal fixation two years ago to correct cervical deformities and a laparoscopic cholecystectomy twenty years ago to address gallstones. The patient did not have any significant family history. The physical examination of the patient was no special. The following laboratory results show abnormal findings: - Red blood cell count: 3.69×10^12^/L (below normal range) - Hemoglobin: 122g/L (below normal range) - Hematocrit: 33.7% (below normal range) - Lymphocyte percentage: 17.6% (below normal range) - Mean platelet volume: 7.8fL - Anion gap: 7.20mmol/L - Albumin: 34.3g/L (below normal range) - Total monocytes: 0.72×10^9^/L (above normal range) - D-dimer: 354.10ug/L (elevated) - Total bilirubin: 28.5umol/L (elevated) - Indirect bilirubin: 24.7umol/L (elevated).

**Figure 1:**
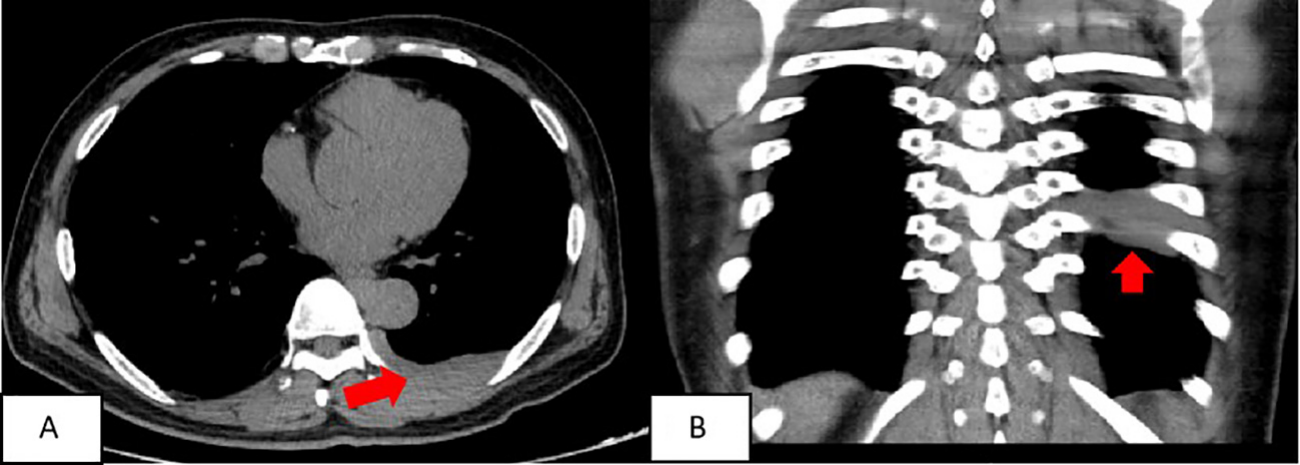
The soft tissue density around the thoracic aorta and the left subpleural area was approximately round or striped, with clear boundaries and uniform density. The larger lesion range was about 5.7cmn1.3cm. **(A)** chest scan transverse position; **(B)** Chest scan coronal position.

**Figure 2:**
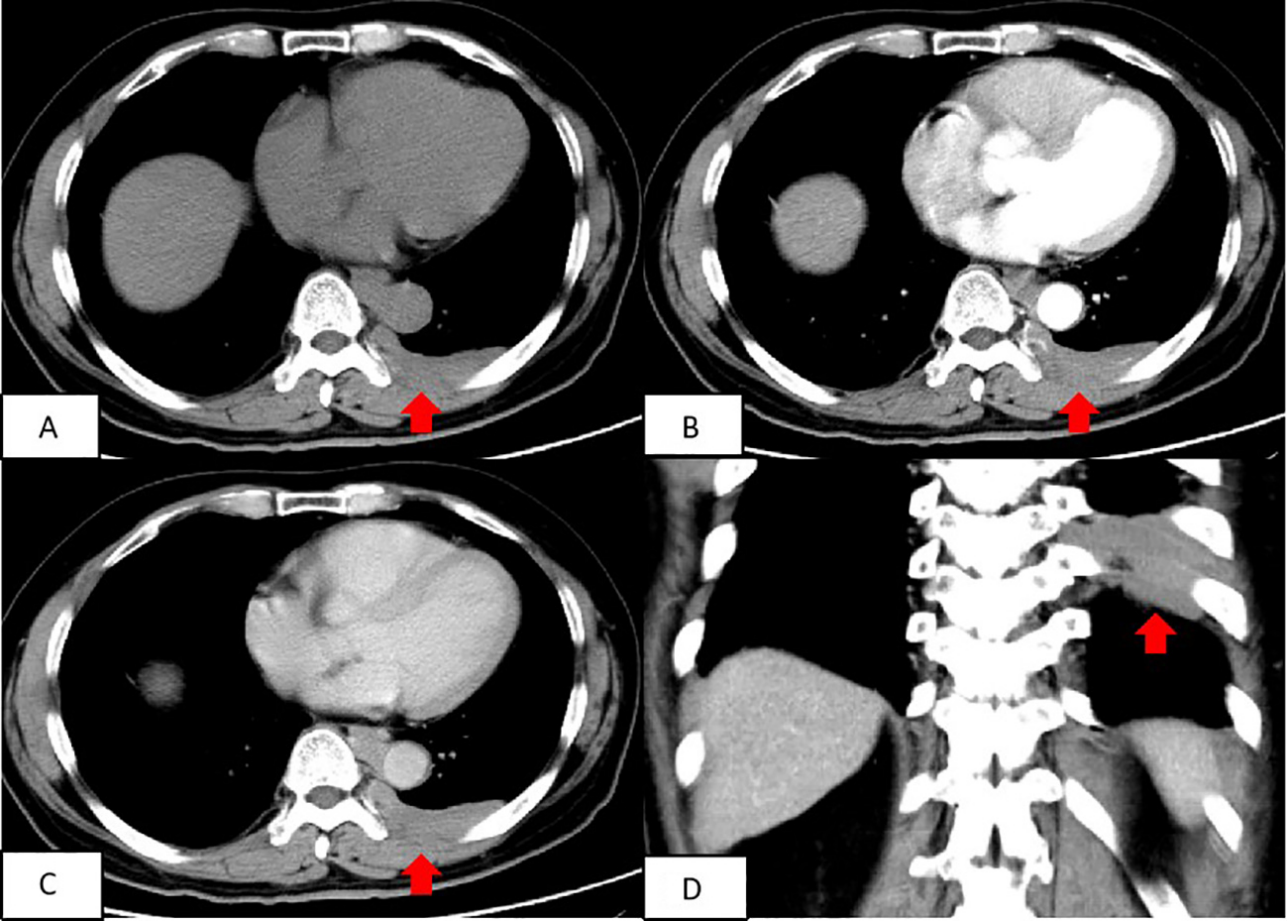
Contrast-enhanced CT scan of the chest depicted a round and flaky soft tissue density shadow in the thoracic aorta and the left lower pleura area, both with uniform density. The size of the lesion was approximately 6.8roximat The shadow was closely associated with the pleura and exhibited mild enhancement on the enhanced scan, with small blood vessel shadows passing through it. **(A)** Transverse position CT plain scan; **(B)** Transection CT scan arterial phase; **(C)** Transection CT scan venous phase; **(D)** Coronal position CT scan.

A chest CT scan revealed soft tissue density shadows in the thoracic aorta and left lower pleural area, with clear boundaries and uniform density. The larger lesion measured approximately 5.7cm×1.3cm and showed no abnormalities in the adjacent ribs (**Figure 1**). Two years later, a follow-up enhanced CT scan showed similar soft tissue density shadows in the thoracic aorta and left lower pleural area, with uniform density and a larger size of about 6.8×1.7cm. These shadows were closely associated with the pleura. The lesion showed creeping growth. The CT value of the lesion was 52.4Hu on plain scan, 64.4Hu on enhanced scan in arterial phase, and 79.9Hu on venous phase. Overall, the lesions showed mild progressive enhancement, and small blood vessel shadows were visible within the lesions. The size of the lesions increased compared with two years ago, and no abnormal density shadows were observed in the adjacent lung tissue (**Figure 2**).

**Figure 3:**
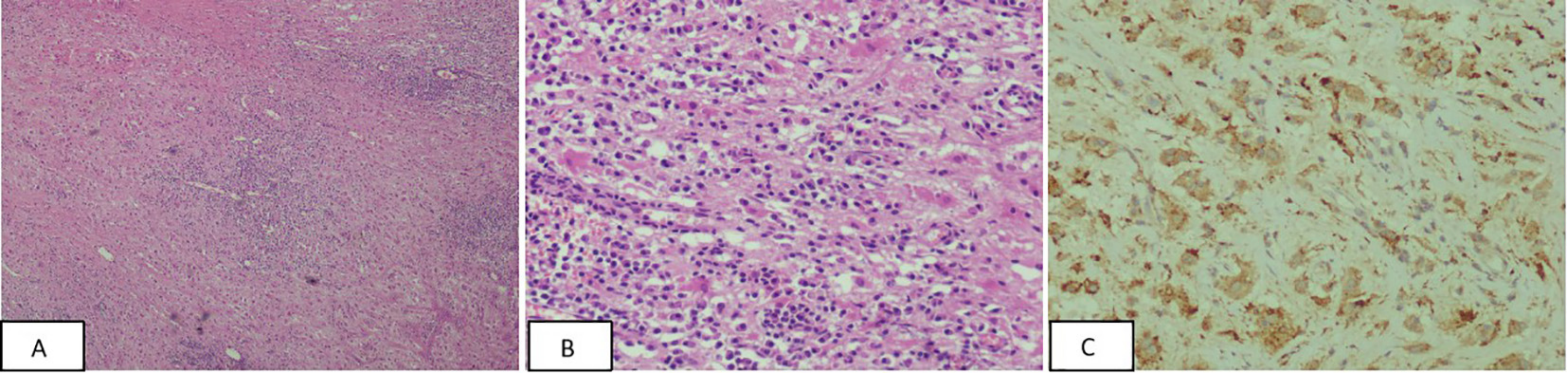
**(A, B)** The pathological image at magnification; cytoplasmic eosinophilic or clear foam-like histiocytes were observed in some areas, and lymphophagocytosis phenomenon was observed [Panel **A** –HE(×40), Panel **B** -HE(×200)]. **(C)** Immunohistochemistry CD68 positive, CD68(×200).

The patient refused a needle biopsy and requested surgical treatment. The patient underwent thoracoscopic resection of left posterior pleural lesions under general anesthesia. No intraoperative pleural effusion or adhesion was observed in the patient’s chest. The interlobar fissure was found to be underdeveloped. A small amount of carbon dust deposition was observed in the lung, but no significant abnormalities were found on the lung surface. The lesion was located in the left posterior chest wall, adjacent to the spine on the lateral side, and the thoracic aorta in the front. It was positioned at approximately the level of the 7-8 posterior ribs, displaying a lamellar prominence with an intact surface envelope and visible neovascularization. Biopsy specimens from the left posterior pleural lesions revealed a large number of plasma cells and lymphocyte infiltration in the fibrous tissue. In some areas, cytoplasmic eosinophilic or clear foam-like histiocytes were observed. Lymphophagocytosis phenomenon was also noted (**Figure 3**). Immunohistochemistry analysis showed the following results: CD1a (-), CD34 (blood vessel +), CD38 (plasma cells +), CD68 (histiocytic cells +), CK (-), igG (partial plasma cells +), igG4 (scattered +), Ki67 (3%+), S100 (+), sox-10 (-), Stat6 (-). Additionally, no BRAF gene V600E mutation was detected in the paraffin-embedded tissue samples collected from the patient.

**Figure 4:**
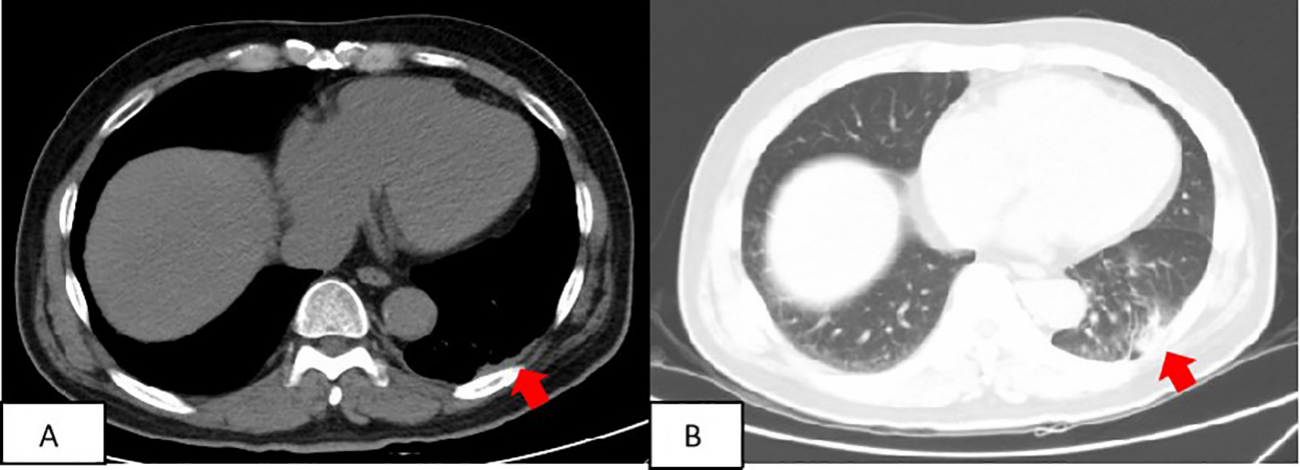
Patient’s follow-up findings: two and a half years after surgery. At the patient’s most recent follow-up, conducted two and a half years after the surgery, a computed tomography (CT) scan revealed a slight thickening of the left pleura **(A)** and mild chronic inflammation in the lung tissue surrounding the surgical site **(B)**.

Based on pathology and immunohistochemistry findings, the definitive diagnosis was pleural primary RDD. The left posterior pleural lesion was surgically resected. Post-operative care included administration of preventive anti-infection medications, measures to reduce phlegm, fluid rehydration, and analgesia. The patients underwent follow-up at 4 months, 1.5 years, and 2.5 years after surgery. Chest CT scans revealed mild thickening of the left pleura and minimal chronic inflammation in the lung tissue neighboring the surgical site (**Figure 4**).

## Discussion

Rosai-Dorfman-Destombes disease (RDD) is a rare non-Langerhans cell histiocytosis. Its main pathological feature is the proliferation of sinus histiocytes, and the complete lymphocyte can be seen in the cytoplasm of some tissue cells, called the lymphophagocytosis phenomenon. RDD most often involves cervical lymph nodes, typically characterized by painless, slow-growing lymph nodes on both sides of the neck in children or young adults (7). In addition, a considerable number of extranodal RDD cases have been reported, including skin, head and neck, chest, spine, and central nervous system (8).

The 2016 revised classification of histiocytosis classifies skin RDD as “Group C” and other RDD as “Group B” (9). The pathogenesis of RDD is still uncertain. At present, it is generally believed that viral infection and immune dysfunction are related to its pathogenesis. Previously, RDD was considered to be a benign non-neoplastic disease. However, recent studies have found that NRAS, KRAS, MAP2K1, and ARAF mutations exist in some RDD lesion tissues, indicating that at least some forms of RDD are neoplastic (10). In the 5th edition of the WHO Classification of Lymphohematopoietic Tumors in 2023, RDD is also included in the chapter on histiocytic/macrophage tumors as a true neoplastic lesion to be explained. The histological feature of RDD is that histiocytic hyperplasia causes lymphatic sinus dilation, surrounded by a large amount of pale cytoplasm, and the boundary is unclear. The presence of intact hematopoietic cells (usually lymphocytes, but also plasma cells, neutrophils, and red blood cells) in the cytoplasm of histiocytes is characteristic of RDD, but extranodal RDD is rare and more often characterized by peripheral fibroplasia, which makes the diagnosis of extranodal RDD difficult. RDD cytology atypia, mitosis, and multinucleation are uncommon. Immunohistochemistry was positive for S100, positive for CD68, and negative for CD1a (11). Negative CD1a can be distinguished from Langerhans cell histiocytosis.

In patients with Rosai-Dorfman disease (RDD), intrathoracic involvement is rare, accounting for only 2% of cases. The most commonly affected sites include the bronchial tree, mediastinal lymph nodes, and thymus, while the involvement of the pleura is very uncommon (12). This particular case is unique as it solely involves the pleura without affecting the lung parenchyma, bronchus, or mediastinal lymph nodes, which is rarely seen in previous reports. Diagnosing pleural RDD through imaging is challenging, as the imaging findings are nonspecific. Three-dimensional CT images of the chest can provide a comprehensive view of the lesion, including changes in its size, shape, position, and surrounding tissue. In this case, a CT scan revealed a thickening of the left pleura with a lamellar soft tissue shadow. The enhanced scan displayed uniform enhancement without invasion of adjacent bone, allowing differentiation from more common pleural malignancies. The Pubmed, Cochrane Library, Web of Science, Scopus, ClinicalTrials.gov and Embase databases were systematically searched for eligible published articles. Through a literature review, we learned that only a few cases of pleural RDD have been reported. Among them, Rathinam A et al. reported a case of pleural-only RDD, describing a 55-year-old female patient with clinical manifestations of chest pain, cough, and CT findings of nonspecific pleural thickening of the left chest wall, which gradually increased and extended along the pleural surface of the upper left inferior lobe over two years (13). This is very similar to our case with pleural lamellar thickening and creeping growth without pleural effusion and hilar and mediastinal lymph node enlargement. Another case of pleural-only RDD describes an 81-year-old female patient with a clinical presentation of shortness of breath and, unlike the two previous cases, mild mediastinal lymph node enlargement or left pleural effusion in addition to left pleural wall thickness (14). Pleural effusion may indicate pleural lymphatic involvement. Deyun Long et al. reported a 49-year-old female patient with clinical manifestations of cough accompanied by right chest pain. CT findings showed multiple nodular soft tissue density masses of different sizes in the right oblique fissure and horizontal fissure of lung and subpleura. The mass density was uniform, and some nodules fused with foliated masses. In this case, the lesions were more extensive, mainly affecting the interlobular pleura. There was also no involvement of both lungs or enlargement of lymph nodes in the neck, mediastinum, or elsewhere (15). Cherif J reported a 58-year-old white male with progressive dyspnea, cough, and left chest pain. Chest radiographs only were available in this case, showing nodular infiltration in the right lower lung field with pleural effusion. Thoracoscopic pleural biopsy indicated RDD (16). Other case reports describe patients with pleural RDD with pleural effusion and concurrent involvement of other serous surfaces, such as the epicardium and mesentery (17, 18).

Currently, the reported cases of pleural RDD, including this one, primarily involve middle-aged and elderly individuals. Common clinical symptoms include cough, chest tightness, chest pain, and shortness of breath. The imaging findings are nonspecific and can be localized pleural lamellar thickening or multifocal, with multiple interlobular pleural nodules fused into masses. With or without pleural effusion, most mediastinal lymph node enlargement is absent. When diagnosing pleural RDD, it is essential to differentiate it from pleural metastatic tumors, pleural mesothelioma, solitary fibrous tumors of the pleura, and schwannoma. The association between RDD and IgG4-related diseases remains controversial. Some studies suggest a link between RDD and IgG4-related diseases (19–21). In this case, immunohistochemistry demonstrated scattered IgG4 expression, indicating a potential association with IgG4-related diseases.

Currently, there is no standardized treatment for RDD, and it must be tailored to the patient’s clinical condition. Asymptomatic skin RDD can be monitored with follow-up and does not necessitate additional intervention. For isolated focal extranodal lesions, such as those in the airways, sinuses, or central nervous system, surgical resection may be performed. In this particular case, the left pleural lesion was surgically removed, resulting in a successful recovery for the patient. Multifocal extranodal lesions that are challenging to remove require systemic therapy, which may consist of sirolimus, corticosteroids, chemoradiotherapy, or immunomodulatory therapy. Targeted therapy can be employed for resistant patients with mitogen-activated protein kinase (MAPK) mutations (22).

## Conclusion

Rarely, RDD may appear to only involve the pleura without affecting the mediastinum and lung tissue. The diagnosis of RDD should be considered when there are radiographic indicators of pleural thickening or multiple nodular, soft tissue lesions in the pleura, with or without pleural effusion. The clinical and imaging manifestations of RDD lack specificity, making it prone to misdiagnosis, especially in the uncommon location of the pleura. A histopathological examination is necessary to confirm the diagnosis. There is no standardized treatment for RDD, as it should be assessed comprehensively based on the lesion site, extent of involvement, and patient’s condition. Treatment plans should be adjusted accordingly during follow-up assessments of therapeutic efficacy.

## Data Availability

The original contributions presented in the study are included in the article/supplementary material. Further inquiries can be directed to the corresponding author.
